# Extracellular vesicles are key intercellular mediators in the development of immune dysfunction to allergens in the airways

**DOI:** 10.1111/j.1398-9995.2010.02359.x

**Published:** 2010-10

**Authors:** T-S Shin, J H Kim, Y-S Kim, S G Jeon, Z Zhu, Y S Gho, Y-K Kim

**Affiliations:** 1 Department of Life Science, POSTECH Biotech Center, Pohang University of Science and Technology (POSTECH)Pohang, Korea; 2 Division of Allergy and Clinical Immunology, Asthma and Allergy Center, Johns Hopkins UniversityBaltimore, MD, USA

**Keywords:** asthma, airway immune dysfunction, extracellular vesicles, lipopolysaccharide

## Abstract

**Background:**

Previous evidence indicates that inhalation of lipopolysaccharide (LPS)-containing with allergens induced mixed Th1 and Th17 cell responses in the airways. Extracellular vesicles (EVs) are nanometer-sized spherical, lipid-bilayered structures and are recently in the public eye as an intercellular communicator in immune responses.

**Objective:**

To evaluate the role of EVs secreted by LPS inhalation in the development of airway immune dysfunction in response to allergens.

**Methods:**

Extracellular vesicles in bronchoalveolar lavage fluids of BALB/c mice were isolated and characterized 24 h after applications to the airway of 10 μg of LPS for 3 days. To evaluate the role of LPS-induced EVs on the development of airway immune dysfunction, *in vivo* and *in vitro* experiments were performed using the isolated LPS-induced EVs.

**Results:**

The inhalation of LPS enhanced EVs release into the BAL fluid, when compared to the application of PBS. Airway sensitization with allergens and LPS-induced EVs resulted in a mixed Th1 and Th17 cell responses, although that with allergens and PBS-induced EVs induced immune tolerance. In addition, LPS-induced EVs enhanced the production of Th1- and Th17-polarizing cytokines (IL-12p70 and IL-6, respectively) by lung dendritic cells. Moreover, the immune responses induced by the LPS-induced EVs were blocked by denaturation of the EV-bearing proteins.

**Conclusion:**

These data suggest that EVs (especially, the protein components) secreted by LPS inhalation are a key intercellular communicator in the development of airway immune dysfunction to inhaled LPS-containing allergens.

Asthma is a very common chronic inflammatory disease that involves the respiratory system, whereby the airways occasionally constrict, become inflamed, and become lined with excessive mucus, often in response to inhaled allergens (1). In terms of the development of asthma, airway immune dysfunction to inhaled allergens is critical for the initiation and orchestration of the inflammatory responses in asthmatic airways (2, 3). The default immune response to the inhalation of innocuous proteins (allergens) is nonresponsiveness (i.e., tolerance) to allergens (4, 5). As inhaled allergens are ubiquitous in the environment, it remains unclear as to why some individuals develop immune dysfunction to allergens, whereas others do not. Household dust allergens are contaminated with lipopolysaccharide (LPS), which is a cell wall component of Gram-negative bacteria and ubiquitously present in the environment (6). Lipopolysaccharide is one of the pathogen-associated molecular patterns (PAMPs) that produce pro-inflammatory and immune-modulating mediators (4, 6). In recent studies, we showed that airway sensitization with LPS-containing allergens induced noneosinophilic inflammation as the result of mixed Th1 and Th17 cell responses to inhaled allergens (7, 8). We also showed that following airway exposure to LPS, the up-regulation of immune-modulating cytokines, such as IL-12p70 and IL-6, plays a key role in the development of Th1 and Th17 polarization, respectively (7, 8).

In multicellular organisms, intercellular communication between cells has been regarded to involve the secretion of soluble factors that subsequently bind to receptors on neighboring cells. Another mode of intercellular communication, the release of extracellular vesicles (EVs), has recently attracted interest (9). Extracellular vesicles are composed of a lipid bilayer that contains transmembrane proteins and encloses soluble hydrophilic components derived from the cytoplasm of the donor cells (9, 10). Depending on cellular sources of EVs, they have been suggested to have diverse immunologic functions; antigen-presenting cell–derived EVs play a role in antigen delivery and T-cell activation, and epithelial cell–derived EVs in tolerance induction (9, 11).

In the present study, we hypothesized that, in addition to the production of soluble mediators, host cells induce immune dysfunction through the secretion of EVs after exposure to LPS-containing allergens. To test this hypothesis, EVs secreted after airway exposure to LPS *in vivo*, in combination with allergens, were applied to the airways of naïve mice. We found that airway exposure of LPS enhanced the secretion of EVs, which were derived from airway epithelial cells and inflammatory cells. Interestingly, we found that airway sensitization with LPS-induced EVs, together with allergens, induced noneosinophilic lung inflammation, which is related to mixed Th1 and Th17 cell responses. Moreover, LPS-induced EVs increased the production levels of both Th1- and Th17-polarizing cytokines (IL-12p70 and IL-6, respectively) by lung dendritic cells (DCs). These results suggest that EVs derived from host cells play a key role in the intercellular communication in the development of airway immune dysfunction to LPS-containing allergens.

## Methods

### Mice

BALB/c mice were purchased from The Jackson Laboratory (Bar Harbor, ME, USA). Mice were bred in the pathogen-free facilities at Pohang University of Science and Technology (POSTECH). All live animal experiments were approved by the POSTECH Ethics Committee.

### Reagents

Lipopolysaccharide was purchased from Calbiochem (La Jolla, CA, USA). Ovalbumin (OVA), polymyxin B (PMB), and Protease K were obtained from Sigma–Aldrich (St. Louis, MO, USA), and phenylmethylsulfonyl fluoride from Bio-Rad Laboratories (Hercules, CA, USA). Antibodies (Abs) used for detecting EV in western blots were: anti-mouse CD81 (Eat-2 clone; Biolegend, San Diego, CA, USA); anti-mouse ICAM-1 (NS0 clone; R&D Systems, Minneapolis, MN, USA); anti-mouse MHC II (IBL-5/22; Santa Cruz Biotechnology, Santa Cruz, CA, USA); anti-mouse surfactant protein-B (SPB02 clone; Abcam, Cambridge, MA, USA). Abs used for FACS analysis were obtained from eBioscience (San Diego, CA, USA).

### Isolation of EVs

**Figure 1:**
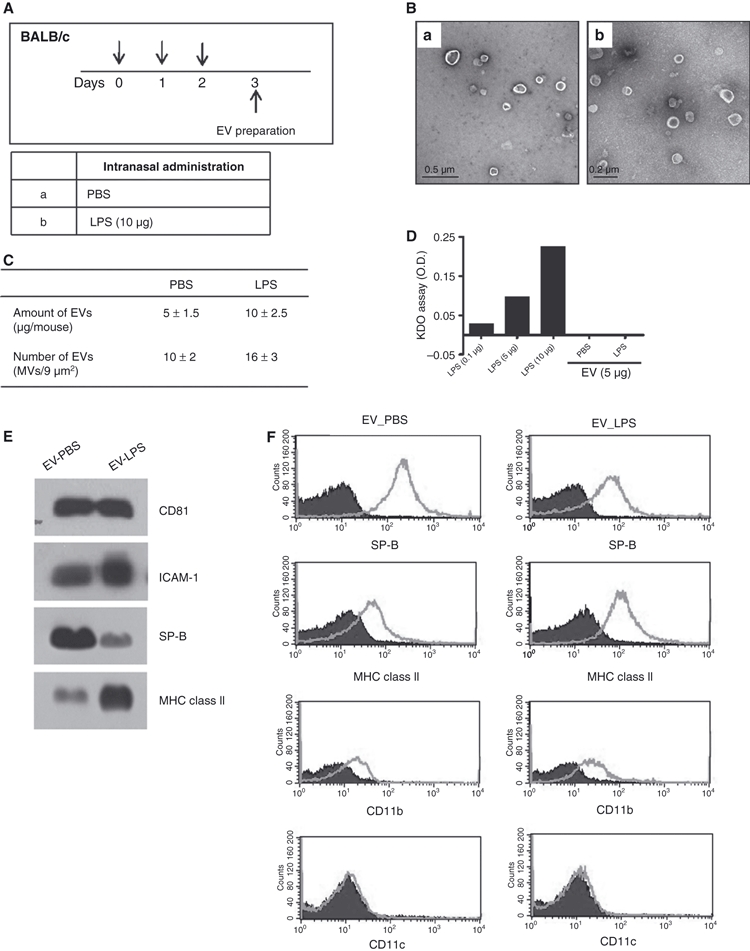
The production of extracellular vesicles (EVs) is enhanced by airway application of LPS, when compared with PBS application. (A) Protocol for EV preparation. (B) Transmission electron microscopy (TEM) images of EVs derived from the Bronchoalveolar lavage (BAL) fluids of PBS-treated (a) and LPS-treated (b) mice (*N* = 20 each group). (C) The amounts and numbers of EVs in the BAL fluids of PBS-treated and LPS-treated mice. The amounts of EVs were quantified on the basis of protein concentrations, as measured using the Bradford assay. The numbers of EVs were determined by counting the EVs in the TEM images. (D) The levels of LPS in the EVs isolated from the BAL fluids of LPS-treated and PBS-treated mice. (E) Western blotting assessment of the levels of host cell marker proteins, CD81, ICAM-1, surfactant protein B (SP-B), and MHC class II, in the EVs derived from LPS-treated and PBS-treated BALB/c mice. (F) FACS data of LPS- and PBS-induced EVs for the expression levels of the airway epithelial cell marker protein surfactant protein B (SP-B) and inflammatory cell marker proteins, MHC class II, CD11b, and CD11c. For (D–F): EV_PBS, EVs from PBS-treated mice; EV_LPS: EVs from LPS-treated mice.

Female BALB/c (6–8 weeks of age) mice were anesthetized with ketamine and xylazine and administrated intranasally with 30 μl PBS with or without LPS (10 μg) on Days 0, 1, and 2 (Fig. 1A). Bronchoalveolar lavage (BAL) fluids were taken 24 h after the last application, and then pooled. The pooled BAL fluids were diluted with PBS, and then centrifuged once at 500 ***g*** for 10 min and once at 3000 *g* for 20 min. To enrich for EVs, the supernatant was loaded onto 0.8 M and 2.5 M sucrose cushions in PBS (pH 7.2) and then subjected to ultracentrifugation at 100 000 ***g*** for 2 h. After centrifugation, the interface of the 0.8 M and 2.5 M sucrose layers was collected and diluted 10-fold in PBS. The sucrose cushion ultracentrifugation procedure was then repeated. The collected interface was diluted 10-fold in PBS and subjected to ultracentrifugation at 100 000 ***g*** for 2 h. Finally, the pellet was resuspended in PBS, and the protein concentration was determined using the Bradford dye assay (Bio-Rad Laboratories, Hercules, CA, USA). EV proteins (2 μg) were analyzed by SDS-PAGE and western blotting or coated beads for FACS analysis, as previously described (11).

### Electron microscopy (EM)

The purified EVs were applied to 400-mesh carbon-coated copper grids (EMS, Matfield, PA, USA). After allowing the EV to absorb for 3 min, the samples were stained with 2% uranylacetate (Ted Pella, Redding, CA, USA), and then electron micrographs were recorded using the JEM 1011 1010 microscope (Jeol, Japan) at an acceleration voltage of 100 kV.

### Limulus amebocyte lysate (LAL) assay to evaluate LPS contamination

To evaluate LPS contamination in the isolated EVs, PBS- and LPS-induced EVs (5 μg) or different doses of LPS, as a standard, were made to react with LAL assay kit (QCL-1000; Lonza, Basel, Switzerland) following an instruction manual. To exclude LPS from the LPS-induced EVs, PMB (10 ng/ml) were pretreated with PBS or LPS-induced EVs (0.5 μg/ml).

### Heat inactivation and pretreatment of protease K

The isolated EVs were incubated at 99°C for 20 min and then treated with chilled ice for 20 min to denaturalize proteins in EVs. To denature surface proteins in EVs, EVs were pretreated with protease K (10 μg/ml) for 15 min and then neutralized with phenylmethylsulfonyl fluoride (1 mM).

### *In vitro* evaluation of EV functions

To evaluate the pro-inflammatory activity *in vitro*, RAW 264.7 cells (3 × 10^5^ cells/well), macrophage cell line, were treated with PBS or LPS (10 ng/ml), as controls, and with PBS- or LPS-induced EVs (0.5 μg/ml). The levels of pro-inflammatory cytokines, such as IL-6 and TNF-α, were quantified from conditioned media 6 h after the incubation.

### *In vivo* evaluation of EV functions

To evaluate the activities of EVs in the development of adaptive immune responses to inhaled allergens, 6-week-old mice were sensitized intranasally with 1 μg PBS or LPS-induced EVs and 75 μg of OVA on Days 0, 1, 2, and 7, and then challenged with 50 μg of OVA alone on days 14, 15, 21, and 22. Allergen-specific adaptive immune responses were evaluated 24 h after the final allergen challenge. Innate immune responses to LPS-induced EVs were evaluated 6 h or 24 h after the sensitization on Day 0.

### BAL cellularity

Bronchoalveolar lavage (BAL) cellularity was analyzed as described previously (7). Briefly, BAL cellularity was determined by counting 300 inflammatory cells after diluting the cell pellets with 50 μl PBS. Inflammatory cells were classified as macrophages, lymphocytes, neutrophils or eosinophils.

### Lung histology

Lung sections were stained with hematoxylin and eosin (H&E) after pressure fixation with Streck solution (Streck Laboratories, La Vista, NE, USA). All sample slides were compared at the same magnification. Lung inflammation was assessed based on the degrees of peribronchiolar and perivascular inflammation, as described previously (12).

### Intracellular staining of lung cells

Cells that were isolated from the lung tissues were incubated in 48-well plates that were coated with anti-CD3 and anti-CD28 antibodies (1 μg/ml each; eBioscience) at 37°C for 6 h. Two hours before the end of incubation, the cells were treated with brefeldin A (10 μg/ml) for 2 h. The cells were stained with surface-specific antibodies (anti-CD3-APC and anti-CD4-FITC; BD Biosciences, San Jose, CA, USA) for 30 min at 4°C, and then fixed in 100 μl fixation solution at room temperature for 20 min. The fixed cells were added with 1 ml permeabilization buffer, centrifuged for 5 min, and then supernatants were aspirated. The cells were incubated with anti-IFN-γ-PE, anti-IL-17-PE, and anti-IL-4-PE antibodies (BD Biosciences) for 30 min at room temperature, and analyzed in a FACScalibur flow cytometer (BD Biosciences) using the cellquest software.

### Isolation of dendritic cells (DCs) and helper T cells and *in vitro* stimulation

Cells that were isolated from lung tissues were stained with microbead-conjugated anti-CD11c or anti-CD4 (Miltenyi Biotec, Auburn, CA, USA), and the CD11c^+^ cells (DCs) or CD4^+^ cells (helper T cells) were sorted using auto-MACS. The isolated DCs were incubated with the isolated EVs in 48-well plates for 6 h; thereafter, the conditioned media were harvested, and then the levels of IL-12p70 and IL-6 were quantified. To evaluate cytokine production from helper T cells, the isolated T cells were incubated in 48-well plates that were coated with anti-CD3 and anti-CD28 antibodies (1 μg/ml each; eBioscience) at 37°C for 6 h, and then the levels of IFN-γ, IL-17, and IL-4 were quantified from conditioned media.

### Cytokine assays

The levels of cytokines in the BAL fluids and cell culture supernatants were measured by ELISA, in accordance with the manufacturer's instructions (R&D Systems, Mineapolis, MN, USA).

### Statistical analysis

Analysis of variance (anova) was used to determine the significance of differences among all the groups. Significant differences among treatments were assessed by using the Student's *t*-test, anova, and Wilcoxon's rank sum test. For multiple comparisons, anova was used initially, and if significant differences were found, individual *t*-tests or Wilcoxon's rank sum tests were performed between pairs of groups. Statistical significance was set at *P* < 0.05.

## Results

### Production of EVs by airway application of LPS

To evaluate the production of EVs following airway application of LPS, 30 μl of PBS with or without 10 μg of LPS were administered intranasally to naïve BALB/c mice, and then the BAL fluids were collected 24 h after the last application (Fig. 1A). The EVs derived from the BAL fluids of the LPS-treated and PBS-treated mice were found to be spherical in morphology, as judged from Transmission electron microscopy (TEM) images (Fig. 1B). The amounts (based on protein concentrations measured using the Bradford assay) and numbers of EVs were higher in the BAL fluids of the LPS-treated mice than in those of the PBS-treated mice (Fig. 1C). The levels of LPS in the EVs isolated from the BAL fluids of LPS-treated and PBS-treated mice were negligible (Fig. 1D). The expression of CD81 and ICAM-1 was detected in the EVs from both groups of mice (Fig. 1E). With regard to the origin of the EVs, the airway epithelial cell marker protein surfactant protein B (SP-B) was expressed in the EVs from both the PBS- and LPS-treated mice; in addition, the expression of an inflammatory cell marker protein, MHC class II, was noted in EVs from both the PBS- and LPS-inhaled mice (Fig. 1E,F). As for the expression of a DC marker protein, CD11c, was absent in EVs from both the PBS- and LPS-inhaled mice, although CD11b, a marker protein of inflammatory cells was expressed in EVs from both the two groups (Fig. 1F). These data together suggest that the secretion of EVs can be inducible by airway exposure to LPS, and that the EVs in the BAL fluid are derived from airway epithelial cells and inflammatory cells other than DCs.

### Lung inflammation induced by airway sensitization with allergens and LPS-induced EVs

**Figure 2:**
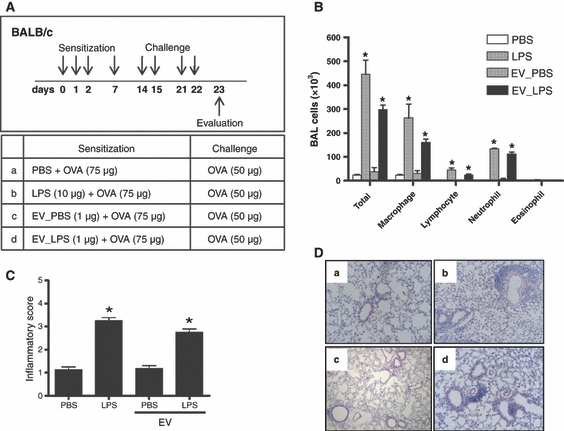
Airway sensitization using allergens with LPS-induced EVs causes noneosinophilic lung inflammation. The evaluation was performed 24 h after the last allergen challenge (*n* = 5 each group). (A) Study protocol. (B) Bronchoalveolar lavage (BAL) cellularity. (C) Inflammatory score, which is based on the peribronchiolar and perivascular infiltration of inflammatory cells in lung histology. (D) Representative lung histology (H&E staining, original magnification ×100). a, PBS; b, LPS; c, EV_PBS; d, EV_LPS. For (B–D) PBS, mice sensitized with PBS plus Ovalbumin (OVA); LPS, mice sensitized with LPS (10 μg) plus OVA; EV_PBS, mice sensitized with PBS-induced EVs (1 μg) plus OVA; EV_LPS, mice sensitized with LPS (10 μg)-induced EVs (1 μg) plus OVA. *P < 0.05 compared to the other groups.

To evaluate the effects of LPS-induced EVs on the development of immune dysfunction to inhaled allergens, naïve BALB/c mice were sensitized with LPS-induced EVs and an allergen (OVA), and then challenged with OVA alone (Fig. 2A). Examination of BAL cellularity showed that infiltration of the lungs by inflammatory cells (macrophages, lymphocytes, and neutrophils, but not eosinophils) was significantly greater in the mice that were sensitized with OVA plus LPS (10 μg) or LPS-induced EVs (1 μg) than in the mice that were sensitized with PBS or PBS-induced EVs (1 μg) (Fig. 2B). The lung inflammatory score, which was based on peribronchiolar and perivascular infiltration of inflammatory cells, was higher in the mice that were sensitized with LPS or LPS-induced EVs than in the mice that were sensitized with PBS or PBS-induced EVs (Fig. 2C). Lung histology revealed that peribronchiolar and perivascular infiltration of inflammatory cells was enhanced in the former two groups, when compared with the latter two groups (Fig. 2D). These findings together suggest that LPS-induced EVs induce airway immune dysfunction to inhaled allergens, although PBS-induced EVs induce immune tolerance.

### Innate immune responses induced by application of LPS-induced EVs to the airways

**Figure 3:**
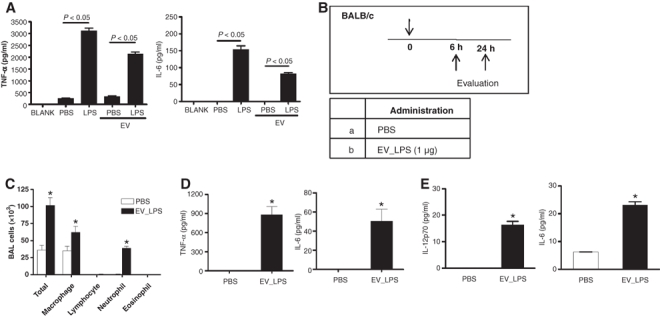
The production levels of pro-inflammatory and immunomodulating cytokines are up-regulated by airway application of LPS-induced EVs. (A) Production levels of TNF-α and IL-6 in the RAW264.7 macrophage cell line after stimulation with PBS, LPS (10 ng/ml), PBS-induced EVs (0.5 μg/ml) or LPS-induced EVs (0.5 μg/ml). (B) Protocol for *in vivo* experiments to evaluate innate immune responses (*n* = 5 each group). (C) BAL cellularity 24 h after airway application of PBS-induced or LPS-induced EVs. (D) *In vivo* production levels of TNF-α and IL-6 at 6 h after airway application of PBS-induced or LPS-induced EVs. (E) *Ex vivo* production of IL-12p70 and IL-6 from lung dendritic cells (DCs) 6 h after stimulation with LPS-induced EVs (0.5 μg/ml). In (C–E) *P < 0.05 compared to PBS group.

The *in vitro* production levels of TNF-α and IL-6 from mouse macrophages (RAW264.7 cells) were enhanced by stimulation with LPS (10 ng/ml) or LPS-induced EVs (0.5 μg/ml), when compared to stimulation with PBS or PBS-induced EVs (0.5 μg/ml) (Fig. 3A). Therefore, we evaluated the effects of LPS-induced EVs on the development of innate immune responses *in vivo*. To test this objective, 1 μg of LPS-induced EVs was applied intranasally to naïve BALB/c mice (Fig. 3B). Bronchoalveolar lavage cellularity 24 h postapplication was significantly increased in the mice that received LPS-induced EVs, when compared to those that received PBS-induced EVs (Fig. 3C). Moreover, the *in vivo* production levels of TNF-α and IL-6 at 6 h postapplication were higher in the mice that received LPS-induced EVs than in those that received PBS-induced EVs (Fig. 3D). Based on the previous data that airway application of LPS-containing allergens induced mixed Th1 and Th17 cell responses *via* the up-regulation of IL-12p70 and IL-6 production, respectively (7, 8), we evaluated the effects of LPS-induced EVs on the production of T-cell polarizing cytokines from antigen-presenting cells. CD11c+ cells (DCs) were isolated from naïve BALB/c mice, and then stimulated with LPS-induced EVs or PBS *in vitro*. The production levels of Th1- and Th17-polarizing cytokines (IL-12p70 and IL-6, respectively) were significantly higher following stimulation with LPS-induced EVs (Fig. 3E). Taken together, these data indicate that EVs secreted in response to LPS cause infiltration of inflammatory cells *via* the up-regulation of pro-inflammatory mediators, such as TNF-α, and modulate adaptive immune responses *via* the up-regulation of Th1- and Th17-polarizing cytokines (IL-12p70 and IL-6, respectively) in antigen-presenting cells.

### The roles of LPS and the protein components of LPS-induced EVs in the development of innate immune responses

**Figure 4:**
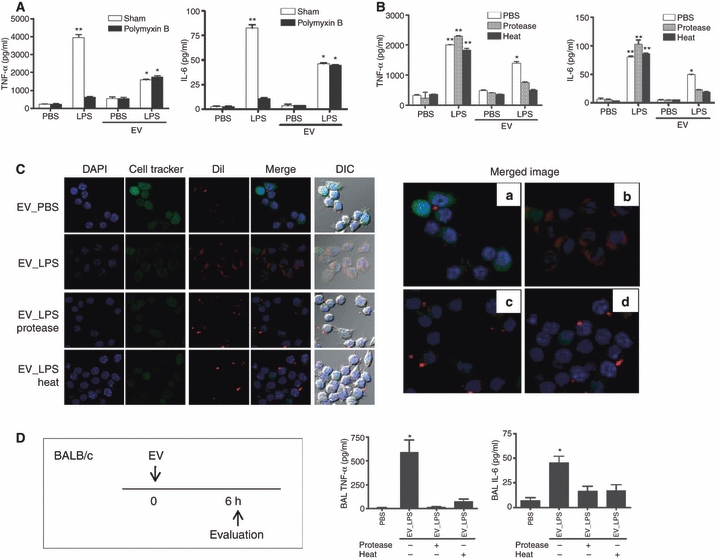
Protein components of the EVs secreted in response to LPS play key roles in the mediation of innate immune responses. (A) The effects of pretreatment with polymyxin B (PMB) (an antagonist of LPS) on the *in vitro* production levels of TNF-α and IL-6 in RAW264.7 macrophages. (B) The effects of pretreatment with protease K or heating on the *in vitro* production levels of TNF-α and IL-6 in RAW264.7 macrophages. For (A and B) *P < 0.05 compared to the EV_PBS group; **P < 0.05 compared to the other groups. (C) Confocal and differential interference contrast (DIC) microscopy imaging of the effects of pretreatment with protease K or heating on the endocytosis of LPS-induced EVs into the cytoplasm of RAW264.7 macrophages. DAPI and cell tracker denote nucleus and cytoplasmic staining, respectively; DiI indicates lipid labeling. In both confocal merged and DIC images show that endocytosis does not occur in LPS-induced EV after pretreatment with protease K or with heat, although it occurred in LPS-induced EVs without these treatments. (a: EV_PBS; b: EV_LPS; c: EV_LPS/protease; d: EV_LPS/heat). (D) Study protocol for *in vivo* experiments (left panel), and the effects of pretreatment of proteinase K or heating on the *in vivo* production of TNF-α and IL-6 in the airways (right panel). *P < 0.05 compared to the other groups.

To evaluate the role of the LPS in the LPS-induced EVs in the innate immune responses, the *in vitro* production levels of TNF-α and IL-6 in lung macrophages were evaluated after incubation with LPS-induced EVs, with or without pretreatment with PMB (an antagonist of LPS). The production levels of TNF-α and IL-6 were similar after stimulation with LPS-induced EVs, with or without pretreatment with PMB, although the LPS-enhanced production of these cytokines was abolished by pretreatment with PMB (Fig. 4A). Taken the absence of any LPS in the LPS-induced EVs into the consideration, this finding indicates that LPS itself in host cell-derived EVs plays no role in the development of the innate immune responses induced by airway LPS exposure.

Next, we evaluated the roles of protein components in LPS-induced EVs. To test this, the host cell–derived EVs were treated with protease K or heating. The production levels of TNF-α and IL-6 from macrophages enhanced by LPS-induced EVs were inhibited by pretreatment with protease K or heating, whereas the production of TNF-α and IL-6 induced by LPS itself was unaffected by these pretreatments (Fig. 4B). Moreover, confocal and differential interference contrast (DIC) microscopy images showed that endocytosis of PBS-induced EVs did not occur, and endocytosis of LPS-induced EVs was blocked by pretreatment with protease K or heating and by PBS-induced EVs, although LPS-induced EVs without pretreatment of protease K or heating were endocytosed into the cytoplasm of the lung macrophages (Fig. 4C). We also evaluated the roles of the protein components in LPS-induced EVs on the *in vivo* production of pro-inflammatory factors. This study showed that the production of TNF-α and IL-6 enhanced by airway application of LPS-induced EVs was abolished by pretreatment with protease K or heating (Fig. 4D). These data together indicate that the protein components in EVs secreted in response to LPS stimulation play key roles in the development of innate immune responses by mediating the endocytosis of signaling factors.

### The role of the protein components in LPS-induced EVs on the development of allergen-specific adaptive responses

**Figure 5:**
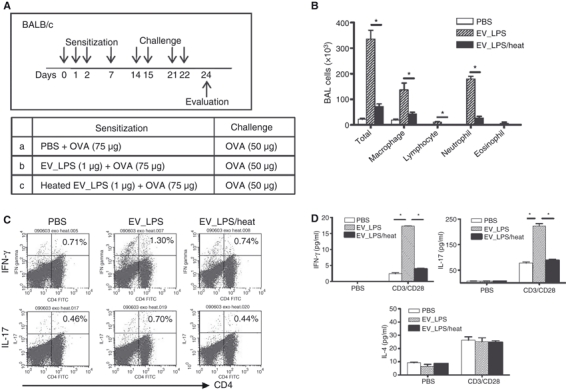
Protein components of EVs secreted in response to LPS are key mediators in the development of mixed Th1 and Th17 cell responses to inhaled allergens. (A) Study protocol for *in vivo* experiments to evaluate adaptive immune responses (*n* = 5 each group). (B) Bronchoalveolar lavage (BAL) cellularity. *P < 0.05. (C) Intracellular staining for Th1 (IFN-γ) and Th17 (IL-17) cytokines: FACS data were derived from CD3^+^ gated lung cells. (D) The levels of Th1 (IFN-γ), Th17 (IL-17), and Th2 (IL-4) cytokines in supernatants 6 h after lung CD4+ T cells were incubated with PBS or anti-CD3 plus anti-CD28 antibodies. *P < 0.05.

Finally, we evaluated the roles of the protein components in LPS-induced EVs on the modulation of adaptive immune responses. To test this, airway allergen sensitization with LPS-induced EVs that were pretreated by heating was performed in naïve BALB/c mice, and the responses were evaluated 24 h after four airway challenges with allergen alone (Fig. 5A). Analysis of BAL cellularity showed that infiltration into the lungs of inflammatory cells (macrophages, lymphocytes, and neutrophils) that were enhanced by sensitization with LPS-induced EVs was significantly inhibited by heat pretreatment of the LPS-induced EVs (Fig. 5B). In terms of the production of T-cell subset cytokines, the numbers of Th1 (IFN-γ) and Th17 (IL-17)-producing T cells in the lungs were increased by sensitization with OVA plus LPS-induced EVs, when compared to sensitization with OVA alone; however, the increases in the numbers of Th1 and Th17 cells were blocked by heat pretreatment of the LPS-induced EVs (Fig. 5C). Moreover, the production levels of IFN-γ and IL-17 from the lung CD4 + T cells, which were increased by sensitization with OVA plus LPS-induced EVs, were significantly inhibited by heat pretreatment of LPS-induced EVs, whereas the production levels of the Th2 cytokine were similar among the three groups (Fig. 5D). Taken together, these findings suggest that the EVs secreted in response to LPS exposure play key roles in the development of allergen-specific Th1 and Th17 cell responses after exposure to LPS-containing allergens, and that the protein components of LPS-induced EVs are key players in the intercellular communication mediated by LPS-induced EVs.

## Discussion

Immune dysfunction can be mediated by host cells through the secretion of EVs, in addition to the production of soluble factors, including cytokines and chemokines (9). Our previous data indicated that airway exposure to LPS-containing allergens induced mixed Th1 and Th17 cell responses and subsequent noneosinophilic lung inflammation (7, 8). In the present study, we show that EVs secreted from host cells, such as airway epithelial cells and inflammatory cells following exposure to LPS *in vivo* induce airway immune dysfunction toward inhaled allergens; this dysfunction is characterized by mixed Th1 and Th17 cell responses that mimic immune responses induced by airway exposure of LPS-containing allergens. To the best of our knowledge, these results are the first report that host cell–derived EVs play a key role in the development of airway immune dysfunction to inhaled allergens.

EVs are spherical structures that are surrounded by a lipid bilayer and contain hydrophilic soluble components (9). EVs can be secreted by budding or shedding from the plasma membranes of platelets (13), tumors (10, 14), neutrophils (15), and DCs (16); these EVs are also referred to as microvesicles, ectosomes, microparticles, and exosomes (9). EVs can also be secreted following the fusion of internal compartments that contain intraluminal vesicles with the plasma membrane; such fusion events, which involve the late endocytic multivesicular bodies (MVBs), were first observed more than 20 years ago (17), and the term exosome has been used to refer to these EVs (18). Electron microscopy studies of tissue sections from tonsil germinal centers have revealed the presence of nanometer-size vesicles that bear MHC II and tetraspanin molecules (19). In addition, EVs have been purified from several body fluids, such as plasma (20), urine (21), and BAL fluid (11, 22). In the present study, we purified EVs from the BAL fluids of mice that were exposed intranasally to LPS or PBS. Our EM and biochemical studies revealed the presence of nanometer-size EVs bearing the tetraspanin molecule CD81, both in the LPS-treated and PBS-treated mice. In addition, the amount of EVs was higher in the LPS-treated mice than in PBS-treated mice, which suggests that EVs can be induced by inflammatory stimuli.

Several types of interaction between EVs and recipient cells have been proposed based on indirect evidence and *in vitro* studies, including adhesion of MVs to the recipient cell surface through lipids or ligand–receptor interactions and the subsequent internalization of whole EVs into endocytic compartments (9). Recent evidence indicates that ICAM-1 in EVs derived from mature DCs are captured by binding to a receptor (lymphocyte function–associated antigen 1, LFA1) on the DC surface (23). Our present data show that EVs secreted in response to LPS in the airways also harbor proteins, including ICAM-1. Moreover, the present confocal and DIC microscopic imaging showed that protein denaturation by pretreatment with protease K or heating blocked the endocytosis of EVs in macrophages, which suggests that protein components in lipid layer of EVs are important for EV endocytosis in recipient cells, in the context of airway immune dysfunction by LPS-induced EVs.

A recent study has shown that tumor-cell-derived EVs can be transferred to T cells, resulting in defective T-cell signaling (24). Our present results show that airway exposure to LPS enhances the release of EVs, and that LPS-induced EVs elicit inflammation and immune modulation through the production of pro-inflammatory and immunomodulatory factors. Moreover, we demonstrate that blockade of EV endocytosis by protein denaturation reverses these deleterious immune responses. These findings suggest that EVs derived from host cells, when exposed to LPS-containing allergens, deliver signaling ligands to recipient cells, e.g., DCs, thereby initiating intracellular signaling in recipient cells to induce innate immune responses that further modulate adaptive responses.

Although there is some evidence indicating that EVs purified *in vitro* affect immune responses *in vivo* (25–27), the roles that EVs secreted *in vivo* modulate immune responses remains to be fully determined. Airway exposure to allergens alone induced immune tolerance; a recent report showed that EVs isolated from the BAL fluids of mice that were tolerized by airway exposure to allergen alone induced immune tolerance to inhaled allergens *via* expression of the regulatory cytokine TGF-β (11). In contrast, our previous data indicated that airway sensitization with allergens in combination with LPS induced mixed Th1 and Th17 cell responses to allergens, which resulted from LPS-induced up-regulation of Th1- and Th17-polarizing cytokines (IL-12p70 and IL-6, respectively) (7, 8) In the present study, we demonstrated that EVs secreted following airway exposure to LPS enhanced the production of both IL-12p70 and IL-6 in lung DCs, as well as subsequent mixed Th1 and Th17 cell responses to inhaled allergens. Moreover, blockade of EV endocytosis by heat-induced protein denaturation reversed the immune dysfunction to inhaled allergens. Taken together, these findings together indicate that EVs represent an important intercellular communicator in the development of immune tolerance or immune dysfunction to inhaled allergens in the airways.

In summary, our present data suggest that EVs secreted by airway exposure to LPS are key players in the development of mixed Th1 and Th17 cell responses to inhaled allergens *via* the up-regulation of Th1- and Th17-polarizing cytokines, and that EVs, in addition to soluble factors, are an important intercellular communication vector between host cells in the development of airway immune dysfunction in the context of inhalation of LPS-containing allergens.
